# Proximity to vector breeding site and risk of *Plasmodium vivax* infection: a prospective cohort study in rural Ethiopia

**DOI:** 10.1186/s12936-017-2031-5

**Published:** 2017-09-19

**Authors:** Alexander Nissen, Jackie Cook, Eskindir Loha, Bernt Lindtjørn

**Affiliations:** 1 Norwegian Centre for Violence and Traumatic Stress Studies, Nydalen, P.O. Box 181, 0409 Oslo, Norway; 2 MRC Tropical Epidemiology Group, London School of Hygiene and Tropical Medicine, London, UK; 3 School of Public and Environmental Health, Hawassa University, Awassa, Ethiopia; 4 Centre for International Health, University of Bergen, Bergen, Norway

**Keywords:** Malaria, *Plasmodium vivax*, Breeding site, Proximity, Distance, Ethiopia, Africa

## Abstract

**Background:**

Despite falling incidence and mortality since the turn of the century, malaria remains an important global health challenge. In the future fight against malaria, greater emphasis will have to be placed on understanding and addressing malaria caused by the *Plasmodium vivax* parasite. Unfortunately, due to years of neglect and underfunding, there are currently many gaps in knowledge of *P. vivax* malaria. The aims of the present study were to explore the association between distance to vector breeding site and *P. vivax* infection in rural Ethiopia, and, secondarily, to test whether this association varies with age.

**Methods:**

A prospective, cohort study of all residents in the Chano Mille Kebele in southern Ethiopia from April 2009 to March 2011 (n = 8121). Weekly household follow up visits included screening for febrile cases (active surveillance). Participants were also asked to contact the local health centre if they experienced subjective fever between visits (passive surveillance). *Plasmodium vivax* infection was confirmed using microscopy by two independent readers. Information was collected on demographics and household characteristics including GPS-determined distance to vector breeding site. Data was analysed using Cox regression modelling.

**Results:**

Overall the *P. vivax* infection rate was 12.3/1000 person-years (95% CI 10.5–14.5). Mean household distance to breeding site was 2449 m (range 1646–3717 m). Fully adjusted results showed very strong evidence of an association between proximity to breeding site and *P. vivax* infection: rate ratio = 3.47 (95% CI 2.15–5.60; P < 0.001) comparing the group closest to the breeding site (distance < 2100 m; n = 1383) to the group furthest away (distance > 2700 m; n = 2460). There was no evidence that age was an effect modifier in the association.

**Conclusion:**

Results showed strong evidence that household proximity to vector breeding site is positively associated with *P. vivax* infection in rural Ethiopia, and that this association is constant across age groups. The findings might influence how net-distribution and indoor residual spraying campaigns are planned, help guide strategies on water resource development by highlighting potential health effects of man-made dams near human habitats, and add to current educational information given to people living close to breeding sites.

## Background

The global burden of malaria has decreased markedly in the last decades due in large parts to increased funding, widespread deployment of insecticide-treated bed-nets, better diagnostics and enhanced availability of artemisinin-based combination therapies [1–3]. Since the turn of the century, malaria incidence worldwide has fallen by more than 60% and 17 countries have eliminated malaria completely, fueling optimism of global malaria eradication [3–7]. There is a growing awareness, however, that eradicating malaria will require a much better understanding of the *Plasmodium vivax* parasite and how to successfully address the challenges it presents. Unfortunately, research on *P. vivax* malaria has lagged far behind research on *Plasmodium falciparum* malaria, and there are currently critical gaps in knowledge across a broad range of topics on *P. vivax* [8–14]. With accumulating evidence that *P. vivax* infection is less benign than once thought [9, 11, 12, 15, 16] and that *P. vivax* prevalence might be underestimated in regions where *P. vivax* and *P. falciparum* coexist [17–19], there is a clear need for further studies.

Even though *P. vivax* malaria primarily affects countries in South and Southeast Asia, *P. vivax* malaria remains an important public health problem around the Horn of Africa [8, 10]. In Ethiopia, more than 50 million people are estimated to live in malaria-risk zones causing between 5 and 10 million clinical infections of malaria per year, and the rate of *P. vivax* infection is comparable to that of *P. falciparum* [20–23]. The number of studies on *P. vivax* malaria in the country has been steadily increasing. However, because vivax malaria is characterized by a high degree of local variation—in part caused by marked regional differences in altitude, temperature and rainfall—knowledge gained from one setting cannot necessarily be transferred to another [24, 25]. Moreover, malaria control programmes have been scaled up markedly in the last decade, meaning results obtained from studies in the early parts of the century or before might be outdated due to changed transmission intensities and dynamics [26, 27].

Various risk factors for *P. vivax* infection have been investigated around the Horn of Africa, including climatic parameters, bed net use, indoor residual spraying, household characteristics and demographic factors [23, 28–41]. A few studies have investigated household proximity to vector breeding site as a risk factor, though the evidence from these studies is mixed: three studies found an enhanced risk [29, 30, 33], one found no difference [28], and one found a reduction in risk with increasing proximity [40]. A separate study concluded that the association between proximity to breeding site and *P. vivax* infection is modified by age, with proximity a much stronger risk factor in children compared to adults [38]. Further research on the topic is warranted.

The aim of the present study was to investigate the association between distance to vector breeding site and *P. vivax* infection in rural Ethiopia, and, secondarily, to examine if age was an effect modifier in the association.

## Methods

### Design, setting and participants

**Fig. 1:**
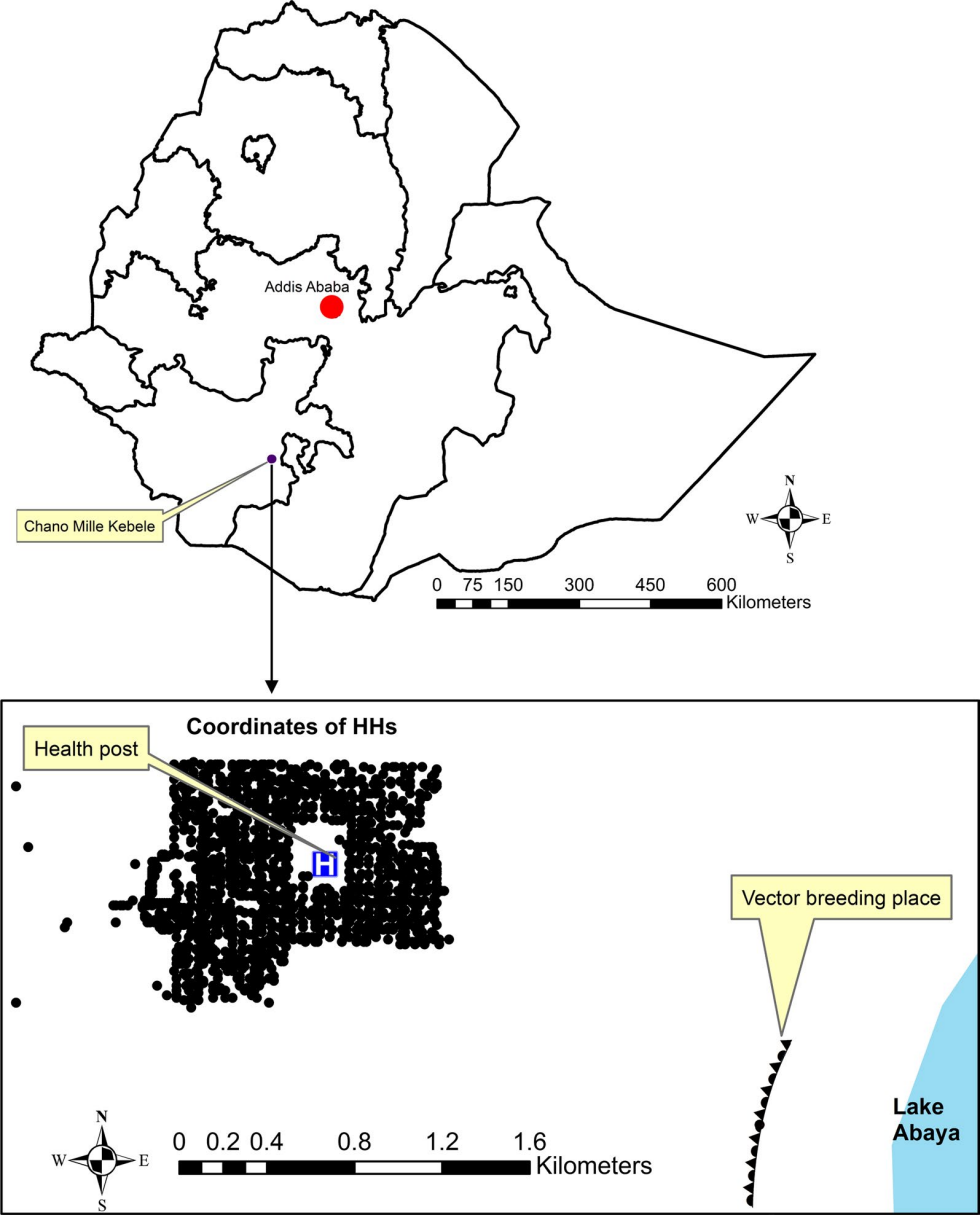
Map of Ethiopia and location of households (dots) in study area (Chano Mille Kebele)

The study used a prospective cohort design and was carried out in the rural Chano Mille Kebele in the Arba Minch Zuria district in Ethiopia, located about 500 km south of the capital Addis Ababa (kebele is the smallest administrative unit in Ethiopia; Fig. 1). The kebele lies about 1200 m above sea-level and covers an area of roughly 2.4 km^2^. The study ran for 101 weeks, from April 2009 to March 2011. The annual level of rainfall was 650 mm in 2009 and 1057 mm in 2010. Three malaria related interventions were carried out over the course of the study period: indoor residual spraying with DDT in May 2009 (week 7) with an estimated coverage of 91%; a free net distribution campaign in March 2010 (week 48) with an average of 2.3 insecticide-treated nets (ITN) given out per household (mean household size was 5.9 persons); and indoor residual spraying with deltamethrin in June 2010 (week 62) with an estimated coverage of 97.5%.

Three censuses were done over the course of the study: in the beginning (week 1); in the middle (week 51) and at the end of the study (week 101). Participants were recruited through the first two censuses. There were no inclusion or exclusion criteria except willingness to participate. All individuals in the Kebele enrolled in the study (i.e. none refused participation), with 7041 enrolling at the first census and 1080 additional individuals at the second census (people who had moved into the Kebele and newborns since the first census were added to the cohort at the second census). The month in which people moved out of the Kebele or died was recorded at the second and third censuses. Information on demographic and socioeconomic variables were collected during all three censuses.

### Active and passive surveillance

The study team visited all participants weekly in their respective households for as long as they were enrolled in the study in order to screen for new cases of *P. vivax* and *P. falciparum* infections and inquire about ITN use (active surveillance). Any febrile participant was sent to the local health care centre for further testing and treatment. The research team double checked at the end of each day that the referred febrile participants had visited the health centre. There were no recorded cases of referred participants failing to come to the health centre. Participants were also instructed to contact the health centre if they experienced subjective fever between the weekly visits (passive surveillance). Further details on methods and results for the *P. falciparum* studies can be found in articles by Loha et al. [36, 42, 43]. Malaria cases detected during the study were treated according to the national guidelines at the time.

All participants were informed about study procedures and gave verbal consent before enrollment. In the case of minors, verbal consent was obtained from caregivers or legal guardians. The study was approved by the Regional Health Research Ethics Review Committee of the Southern nations, nationalities and People’s Regional Health Bureau.

### Data and analysis

Clinical episodes of *P. vivax* infections were detected through either active or passive surveillance. The following two criteria defined a case: (i) Axillary temperature ≥ 37.5 °C during a weekly household visit (active surveillance) or subjective fever experienced between visits resulting in participants contacting the health centre (passive surveillance); and (ii) positive microscopy reading by two of three experienced readers using standardized WHO procedures [44].

Potential vector breeding sites were investigated by tracking larvae of the Anopheles species, and the only place where larvae were found was at a swampy area close to Lake Abaya. During the rainy season, the area contains many small bodies of water formed as hoof-prints from cattle and hippopotami fill with water when the lake floods. The identified breeding site is located about 2.5 km south-east of the centre of the Kebele. The position of each household was determined using global positioning systems coordinates, enabling the distance from the household to the perimeter of the breeding site to be estimated to within 5–10 m accuracy (see Fig. 1). In order to simplify analysis and test for interaction, household proximity to breeding site was categorized into the following four groups: > 2700 m (used as reference category); 2400–2700; 2100–2400; and < 2100 m. Cut-off points were chosen with the aim of having evenly spaced groups with comparable number of participants in them.

Information on age, gender, persons living in household, education of head of household, and household wealth estimated through a wealth index score [42] was collected for all participants during the first two censuses. Starting from week 5 of the study, all participants were asked during the weekly household visits whether they slept under an insecticide treated net (ITN) the night before the visits. ITN use fraction was calculated as the number of nights reportedly sleeping under a net divided by the total number of nights asked. Because ITN use increased substantially in the Kebele following the net distribution campaign at week 48, the follow-up period was split in two at this point. ITN use fraction was then calculated for the complete follow-up period as well as for the two periods before and after the campaign. For participants experiencing a *P. vivax* infection, ITN use fraction was calculated only for weeks prior to infection.

Participants experiencing a *P. vivax* infection exited the study at the time of their infection. For participants with multiple *P. vivax* infections, therefore, only the first episode was counted. The reasoning behind this was twofold. First, a fairly high proportion of reinfections with *P. vivax* might in fact represent relapses of the dormant hypnozoite stage of *P. vivax* rather than new infections from new mosquito bites [45–48]. Second, participants experiencing a *P. vivax* infection would probably increase their use of ITN as a result of the infection, and this might distort results.

T tests and Chi square tests were used evaluate potential selection bias in the groups lost to follow-up and to compare participants entering the study at the second census to participants entering at the beginning on the following parameters: distance to breeding site; age; gender; and ITN use. Fischer’s exact test was used if there were few individuals in an exposure category, and non-parametric tests if variables deviated substantially from normality. The Mantel–Haenszel method was used to obtain unadjusted *P. vivax* rate ratios (RR), and to do a preliminary confounder evaluation by comparing crude to confounder-adjusted associations of increasing proximity to breeding site with *P. vivax* infection rates, adjusting for one confounder in turn. Lexi’s expansion was applied to test if *P. vivax* infection rates differed before and after the net distribution campaign.

Multivariable analysis was done with Cox regression modelling. The proportional hazards assumption was tested both through visual inspection of a log-scaled Nelson Aalen plot and a more formal hypothesis test of proportional hazards using likelihood ratio test (LRT). The Cox regression model was built with a forward, stepwise approach, with potential confounders added one at a time to the basic model containing only distance to breeding site. Gender and age were added first for a priori reasons. The other variables were added based on the strength of confounding found in bivariable analysis and kept in the model if they improved overall fit or were confounders in the multivariable model. Likelihood ratio test (LRT) was used to compare models, test for interaction and test for departure from linear trend for distance to breeding site. The best-fitted Cox regression model without adjustments for ITN use fraction was then compared to a model where use fraction for the whole period was included. Finally, the follow-up period was split in two at the point of the net distribution campaign (week 48) so that the effect of ITN use fraction could be investigated separately for the two periods.

## Results

### Flow of participants through the study

**Fig. 2:**
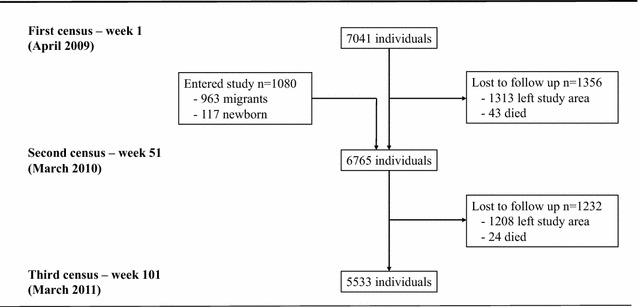
Flowchart of participants through study, Chano Mille Kebele, Ethiopia, April 2009 to March 2011 (n = 8121)

A total of 7041 individuals enrolled at study start. Figure 2 summarizes the flow of participants through the study. None of the eligible participants refused to take part in the study, therefore, the population at study start consisted of all individuals living in the Kebele in April 2009. The percentage lost to follow-up between the first and second census was 19.3 (1356 of 7041 participants), and the percentage lost between the second census and the end of the study was 18.3 (1232 of 6765 participants). The group lost in the first period had a higher proportion of males, was less likely to use bed nets regularly, and lived closer to the breeding site compared to those who remained in the study. Similarly, the group lost in the second period had a higher proportion of males and was less likely to use bed nets regularly compared to those who remained in the study until the end, however, the group lived further away from the breeding site. Participants entering the study at the second census were younger and lived further away from the breeding site on average compared to participants entering at the beginning.

### Descriptive results and preliminary analysis

**Fig. 3:**
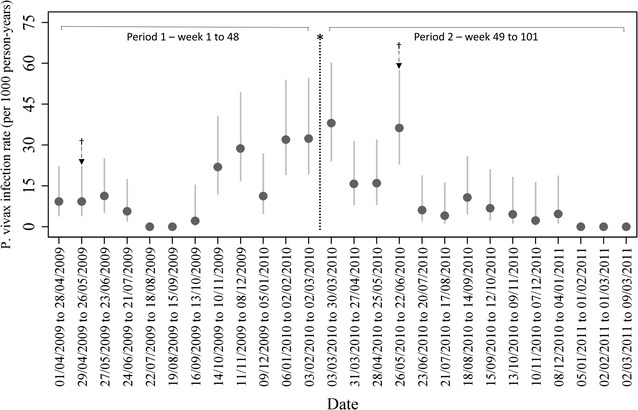
*Plasmodium vivax* infection rates across study period per 1000 person years (95% CI). Asterisk: Net distribution campaign (week 48). Dagger: Indoor residual spraying

There were 193 *P. vivax* episodes in 146 participants over the course of the study. Of these 146 participants, 112 experienced one episode, 24 experienced two episodes, seven experienced three episodes and one experienced four episodes. If all episodes were counted (i.e. participants were not excluded upon first infection), the overall *P. vivax* infection rate was 15.8/1000 person-years (95% CI 13.7–18.2). If repeat infections were excluded, the overall *P. vivax* rate was 12.3/1000 person-years (95% CI 10.5–14.5). The average follow-up time for the whole cohort was 76.3 weeks (n = 8121), and the average time to first infection was 40.6 weeks (n = 146). The average follow-up time for participants entering at study start (n = 7041) and after the second census (n = 1080) was 81.7 and 41.3 weeks, respectively, and the average time to first infection for the two groups was 44.3 (n = 131) and 8.1 weeks (n = 15). Figure 3 summarizes *P. vivax* infection rates for 4-week periods over the course of the study.

Participants characteristics and univariable associations are summarized in Table 1. The average distance form participants’ households to mosquito breeding site for the whole cohort was 2449 m with a range of 1646–3717 m. The 25th, 50th and 75th‰ were 2215, 2524 and 2738 m, respectively.

**Table 1:** Baseline characteristics across risk factors and unadjusted associations between risk factors and vivax infection rates

Participant characteristics	No. of individuals (%)	No. with infection	Follow-up (per-years)	Rate/1000 per-years	Rate ratio (RR)	95% conf. interval	P value^c^
Household distance to breeding site (n = 8121), m							
> 2700	2460 (30.3)	26	3670.0	7.08	Ref		
2400–2700	2527 (31.1)	33	3691.5	8.94	1.26	0.76–2.11	0.37
2100–2400	1751 (21.6)	38	2472.5	15.37	2.17	1.32–3.57	0.002
< 2100	1383 (17.0)	49	2044.1	23.97	3.38	2.10–5.44	< 0.001
Age at study entry (n = 8121), years							
≥ 25	2558 (31.5)	16	4056.8	3.94	Ref		
15–24	2321 (28.6)	19	2732.8	6.95	1.76	0.91–3.43	0.09
5–14	2175 (26.8)	69	3435.4	20.08	5.09	2.96–8.77	< 0.001
0–4	1067 (13.1)	42	1653.1	25.41	6.44	3.62–11.46	< 0.001
Gender (n = 8121)							
Male	4227 (52.1)	82	6045.4	13.56	Ref		
Female	3894 (47.9)	64	5832.7	10.97	0.81	0.58–1.12	0.20
Household wealth (n = 8121)							
1 = wealthiest tertile	2282 (28.1)	45	3177.5	14.16	Ref		
2	3079 (37.9)	47	4599.3	10.22	0.72	0.48–1.09	0.12
3 = poorest tertile	2760 (34.0)	54	4101.3	13.17	0.93	0.63–1.38	0.72
Education head of household (n = 8121)							
Secondary or above	1720 (21.2)	26	2574.8	10.10	Ref		
Primary	2050 (25.2)	43	3014.7	14.26	1.41	0.87–2.30	0.16
No education	4351 (53.6)	77	6288.6	12.24	1.21	0.78–1.89	0.39
Persons living in household (n = 8121)^a^							
≤ 7	4445 (54.7)	73	6619.1	11.03	Ref		
≥ 8	3676 (45.3)	73	5259.0	13.88	1.26	0.91–1.74	0.16
Time-period							
Period 1 (week 1–48)	–	76	5797.7	13.11	Ref		
Period 2 (week 49–101)	–	70	6080.4	11.51	0.88	0.64–1.22	0.43
ITN use fraction^b^							
Complete follow-up period (n = 8096) (%)							
> 50	2878 (35.6)	32	4925.0	6.50	Ref		
≤ 50	5218 (64.4)	109	6952.2	15.68	2.41	1.63–3.58	< 0.001
Period 1 (week 1–48) (n = 7016) (%)							
> 50	1314 (18.7)	16	1167.5	13.70	Ref		
≤ 50	5702 (81.3)	55	4629.3	11.88	0.87	0.50–1.51	0.62
Period 2 (week 49–101) (n = 6695) (%)							
> 50	4163 (62.2)	34	4029.0	8.44	Ref		
≤ 50	2532 (37.8)	36	2051.4	17.55	2.08	1.30–3.32	0.002

^a^The number of persons per household ranged from 1 to 17, with a mean of 5.9 and a median of 7 (used as the cut-off point)

^b^ITN use was recorded weekly (starting from week 5) during the regular follow-up visits to households. Participants were asked if they slept under an ITN the night before the visit. Use fraction was calculated as the number of nights reportedly sleeping under a ITN divided by the total number of nights asked. At week 48, there was a net distribution campaign in the Kebele, thus use fraction is presented both for the complete follow-up period as well as with the follow-up period split in two. For those with *P. vivax* infection, ITN use fraction was calculated only for weeks prior to infection

^c^P value from the Mantel–Haenszel test of RR = 1.00

Unadjusted analysis showed strong evidence that increasing proximity to breeding site was associated with elevated rates of *P. vivax* infection. If modelled linearly, the rate of *P. vivax* infection increased with more than 50% per one-category increase in proximity (RR = 1.54, 95% CI 1.32–1.79; LRT of no departure from linearity: P = 0.77). There was also strong evidence that younger participants had higher rates of *P. vivax* infection than older participants, and that participants sleeping under a ITN ≤ 50% of nights had increased rates of infection compared to those sleeping under a ITN > 50% of nights. There was weak or no evidence that the other covariates were associated with *P. vivax* infection, making them unlikely confounders. There was no evidence that the overall rate of *P. vivax* infection went down following the net distribution campaign during week 48, however, a much higher percentage of participants reportedly used bed nets more than half the nights: 19 versus 62% for the period before and after the net campaign, respectively.

### Cox regression models

Model 1, Table 2, shows the best-fitted Cox regression model without adjustments for ITN use. After adjusting for age at study entry, gender, persons living in household, education head of household and household wealth, there was strong evidence of increasing rates of *P. vivax* infection the closer a participant lived to the breeding site (*P. vivax* rates increased by 50% for each one-category increase in proximity to breeding site). If the group closest to the breeding site was compared to the group furthest away, the closest group had almost 3.5 times the rate of *P. vivax* infection (RR = 3.47, 95% CI 2.15–5.60, P < 0.001). None of the covariates confounded the association between distance to breeding site and *P. vivax* infection. LRT of age as an effect modifier between distance to breeding site and *P. vivax* infection gave no evidence of interaction. There was minimal change in the association between proximity to breeding site and *P. vivax* infection when ITN use fraction for the whole follow-up period was added (RR = 1.56, 95% CI 1.34–1.81, P < 0.001, Table 2, Model 2), and there was no evidence that age was an effect modifier. Finally, there was no evidence against modelling proximity to breeding site linearly.

**Table 2:** Cox regression models with adjusted rate ratios of *P. vivax* infection for various risk factor levels

	Model 1—no adjustments for ITN use fraction (n = 8121)		Model 2—adjusted for ITN use fraction (n = 8096)	
	RR (95% CI)	P value	RR (95% CI)	P value
Increasing household proximity to breeding site^a^	1.50 (1.30–1.74)	< 0.001	1.56 (1.34–1.81)	< 0.001
Age at study entry				
≥ 25	Ref		Ref	
15–24	1.73 (0.89–3.36)	0.11	1.40 (0.69–2.83)	0.35
5–14	4.71 (2.73–8.13)	< 0.001	3.54 (1.95–6.43)	< 0.001
0–4	6.49 (3.64–11.55)	< 0.001	5.47 (2.98–10.06)	< 0.001
Gender				
Males	Ref		Ref	
Females	0.85 (0.61–1.18)	0.34	0.86 (0.62–1.20)	0.38
Persons living in household				
≤ 7	Ref		Ref	
≥ 8	1.27 (0.92–1.77)	0.15	1.16 (0.83–1.63)	0.39
ITN use fraction (%)^b^				
> 50			Ref	
≤ 50			1.85 (1.20–2.83)	0.005

^a^Distance to breeding site was categorized into: > 2700 m (ref); 2400–2700; 2100–2400; and < 2100 m, and modelled linearly

^b^ITN use was recorded weekly (starting from week 5) during the regular follow-up visits to households. Participants were asked if they slept under a ITN the night before the visit. Use fraction was calculated as the number of nights reportedly sleeping under a ITN divided by the total number of nights asked. For those with *P. vivax* infection, ITN use fraction was calculated only for weeks prior to infection

Secondarily, there was strong evidence that age was negatively associated with *P. vivax* infection (the age group 0–4 years had more than 5 times the rate of infection compared to the age group ≥ 25 years); and that ITN use fraction was negatively associated with infection rates (participants sleeping under a ITN 50% of nights or less had 85% higher rates of *P. vivax* infection). ITN use also appeared to be a positive confounder of the association between age and *P. vivax* infection, with adjusted RRs being pushed closer to the null of RR = 1.00 across all age groups.

When the follow-up period in the fully adjusted model was split at the point of the net distribution campaign, the strength of the association between distance to breeding site and *P. vivax* infection was about 10% greater for the first time-period, though confidence intervals overlapped (RR = 1.60, 95% CI 1.29–1.98 before net campaign versus RR = 1.46, 95% CI 1.17–1.81 after net campaign; Table 3). There was no evidence that age was an effect modifier in either period, and there was no evidence against modelling proximity to breeding site linearly.

**Table 3:** Fully adjusted Cox regression models with follow-up period split at net distribution campaign

	Period 1—week 1 to 48 (n = 7016)		Period 2—week 49 to 101 (n = 6695)	
	RR (95% CI)	P value	RR (95% CI)	P value
Increasing household proximity to breeding site^a^	1.60 (1.29–1.98)	< 0.001	1.46 (1.17–1.81)	0.001
Age at study entry				
≥ 25	Ref		Ref	
15–24	2.73 (0.88–8.47)	0.08	1.23 (0.50–3.00)	0.65
5–14	9.87 (3.79–25.70)	< 0.001	2.43 (1.15–5.13)	0.02
0–4	10.79 (4.03–28.92)	< 0.001	4.57 (2.13–9.79)	< 0.001
Gender				
Males	Ref		Ref	
Females	0.79 (0.49–1.27)	0.33	0.96 (0.60–1.53)	0.85
Persons living in household				
≤ 7	Ref		Ref	
≥ 8	0.83 (0.52–1.35)	0.46	1.81 (1.11–2.97)	0.02
ITN use fraction (%)^b^				
> 50	Ref		Ref	
≤ 50	0.67 (0.37–1.20)	0.18	1.68 (1.01–2.79)	0.04

^a^Distance to breeding site categorized into: > 2700 m (ref); 2400–2700; 2100–2400; and < 2100 m, and modelled linearly

^b^ITN use was recorded weekly (starting from week 5) during the regular follow-up visits to households. Participants were asked if they slept under a ITN the night before the visit. Use fraction was calculated as the number of nights reportedly sleeping under a ITN divided by the total number of nights asked. During week 48, there was a net distribution campaign in the Kebele thus the follow-up period was split at this point. For those with *P. vivax* infection, ITN use fraction was calculated only for weeks prior to infection

Secondarily, there was some indication that younger age was a stronger risk factor for *P. vivax* infection in the first period when the overall ITN use fraction in the population was lower compared to in the second period.

## Discussion

This 2-year prospective cohort study conducted between 2009 and 2011 in the rural Chano Mille Kebele in southern Ethiopia examined the association between *P. vivax* infection and household distance to vector breeding site using Cox regression modelling. The study found strong evidence of increasing rates of *P. vivax* infection the closer participants lived to the breeding site after adjusting for age, gender, persons living in household, education head of household, household wealth and ITN use. The final regression model showed that the group of participants who lived closest to the breeding site had almost 3.5 times the rate of *P. vivax* infection compared to the group who lived furthest away. The effect of increasing proximity to breeding site on *P. vivax* infection rate appeared to be linear throughout the distance categories investigated. There was no evidence that age was an effect modifier, investigated as a secondary objective in the study. Other important, though post hoc, findings included strong evidence that age was negatively associated with *P. vivax* infection (rates were more than 5 times higher in the age group 0–4 years compared to adults 25 years or older), and that overall ITN use was associated with reduced *P. vivax* infection rates. There was also some indication that the increased rate of *P. vivax* infection associated with younger age was more pronounced in the time-period when overall reported ITN use was low in the population (i.e. before the net distribution campaign).

The main finding of the present study, that there was a clear positive association between household proximity to vector breeding site and *P. vivax* infection, is in line with the study’s pre-specified hypothesis and consistent with the majority of evidence from similar research conducted around the Horn of Africa [29, 30, 33, 38]. Loha et al. also found a positive association between proximity to breeding site and *Plasmodium falciparum* infection in the same study population [42]. Given how malaria is transmitted, it is biologically plausible that individuals in households close to where mosquitoes live and breed have increased risk of infection [16]. The female anopheline mosquito carrying the malaria parasite requires a blood-meal after mating in order for eggs to develop, and as the female leaves the breeding site in search of a suitable human host, it is logical that individuals who are closer to where the search starts have a higher likelihood of being targeted. However, it is not necessarily household proximity to breeding site per se that matters, rather, household proximity might be considered a strong positive correlate of the relative probability of being bitten and infected. This probability also depends on many other factors. For example, Loha et al. found evidence that the risk of malaria infection for individuals in a given household at a given distance to the breeding site was reduced if there were many other households lying between their household and the breeding site [36]. In other words, the spatial layout of households in a village in relation to the breeding site might influence malaria infection risk and modify the effect of proximity in and of itself. Similarly, it is possible that other physical characteristics of a village or region—e.g. topography or vegetation—might modify the effect of proximity to breeding site.

The strength of the findings is supported by the clear stepwise increase in the rate of *P. vivax* infection with increasing proximity to breeding site. The evidence of linearity between proximity and *P. vivax* infection is somewhat at odds with findings from another study in Ethiopia where the relationship tended be exponential [34]. Importantly though, the distance range investigated in that study was 150–1250 m compared to 1646–3717 m in the present study. It is possible that *P. vivax* infection rates increase exponentially with proximity to breeding site at distances close to the breeding site and more linearly at distances further away.

As discussed in the limitation section below, it is hard to exclude selection bias resulting from loss to follow-up. However, when the follow-up period was split at the point of the net distribution campaign, the evidence of an association between proximity to breeding site and *P. vivax* infection was strong for both periods even though the population in the two periods differed (due to both follow-up loss and the entry of new participants). This makes it less likely that selection bias has greatly distorted the main conclusion. Misclassification and residual confounding can also not be excluded.

In terms of the study’s secondary objective of investigating age as an effect modifier, there was no evidence neither in bivariable nor multivariable analysis to support this. This finding contrasts an earlier study by Peterson et al. which found the effect of proximity to breeding site to be more pronounced in younger age groups [38]. Several reasons might explain this difference. One, the present study was conducted in a rural setting at an altitude of about 1200 m versus the study by Peterson et al. which was done in a peri-urban setting at an altitude of 1600 m. Another important difference is that the study by Peterson et al. was of shorter duration and conducted during peak malaria season with consequently higher incidence rates of malaria over the course of the study period (the present study covered both peak and non-peak season). Though perhaps the most important differences between the studies were that the overall ITN use fraction was much lower in the study by Peterson et al. and that the distance-range investigated was much closer to the breeding site.

The post hoc finding that younger age was strongly associated with higher rates of *P. vivax* infection is in line with prior research [30, 37, 39], and in accordance with current understanding of the development of *P. vivax* immunity [49]. Since immunity against *P. vivax* develops at an earlier age than immunity against *P. falciparum,* is was unsurprising that the youngest age group had significantly higher *P. vivax* rates compared to the second youngest group in the present study, however, that *P. falciparum* rates in the same population were roughly equal in the two age groups [42]. The tendency that younger age was a much stronger risk factor for *vivax* infection in the time period before the net distribution campaign should be interpreted with caution given the large and overlapping confidence intervals. The might be a topic of interest for future studies.

The study did not find evidence that gender, household wealth or education of head of household were associated with *P. vivax* infection, which contrasts conclusions from other studies [28, 31, 37, 40, 41]. Part of this discrepancy might be because the present study lacked power to detect these associations (versus Siri [41]); categorized variables differently (versus Graves et al. and Khaireh et al. [28, 31]; or required objective/subjective fever in addition to a positive RDT and microscopy reading to meet *P. vivax* case definition (versus most other studies which did not require clinical signs or symptoms [28, 31, 37, 41]. Lastly, given that the transmission of *P. vivax* depends on many setting specific characteristics (e.g. altitude, rainfall, temperature), it might well be that a given variable is an important risk factor in one setting, though less important in another setting with different transmission patterns.

### Study limitations

Loss to follow-up is a potentially important limitation to consider. However, because there was inconsistent evidence on how loss to follow-up was associated with proximity to breeding site, and because the design allowed for new participants midway through the study, it is difficult to evaluate how loss to follow-up might have affected results. There was consistent evidence that those lost to follow-up were younger and had a lower proportion with ITN use over 50% which would lead to an underestimation of the overall rate of infection in the study (the age difference was small in absolute terms, thus the likely biological effect of this should be limited). Since those lost to follow-up in addition had a higher proportion of males, care should be taken when generalizing to populations based on similar baseline characteristics.

The diagnosis of *P. vivax* with microscopy is not straightforward in settings where *P. falciparum* and *P. vivax* coexist [9, 17, 19], which could result in under diagnosis and/or misclassification of *P. vivax* malaria (non-differential). In terms of ITN use, it is possible that reported use the night before the weekly visits was too imprecise to adequately control for ITN as a confounder, resulting in residual confounding. However, a strength of the study was the thoroughness of the data collection on ITN use compared to most other studies on *P. vivax* malaria in the region. Residual confounding might also result from dichotomizing the ITN variable.

The decision that participants experiencing *P. vivax* infection would exit the study at the point of their first infectious episode might also be considered a limitation. That is, by excluding the contributed follow-up time after infection for these participants, the final model used less information than was available in the data-set. Nonetheless, because ITN use patterns likely changed for participants as a result of being infected, and because repeat infections might be relapse episodes of the dormant hypnozoite stage of the *P. vivax* parasite rather than truly new infections [45–48], excluding participants upon their first episode was deemed the best approach.

### Conclusion and recommendations

The present study found strong evidence for a positive association between *P. vivax* infection rates and living close to a vector breeding site. Contrary to earlier research, there was no evidence that this association varied across age groups. The transmission dynamics of *P. vivax* malaria depend on many factors, some of which are highly setting specific—e.g. climate and altitude. Therefore, care should be taken when trying to apply the findings from the present study to populations in different settings.

The findings might influence how net-distribution and indoor residual spraying campaigns are planned and implemented, help guide strategies on water resource development by highlighting potential negative health effects of man-made dams near human habitats, and add to current educational information given to people living close to vector breeding sites.

## References

[1] Cotter C, Sturrock HJW, Hsiang MS, Liu J, Phillips AA, Hwang J (2013). The changing epidemiology of malaria elimination: new strategies for new challenges. Lancet.

[2] Hemingway J (2014). The role of vector control in stopping the transmission of malaria: threats and opportunities. Philos Trans R Soc Lond B Biol Sci.

[3] WHO (2016). World malaria report: summary.

[4] Tanner M, Greenwood B, Whitty CJM, Ansah EK, Price RN, Dondorp AM (2015). Malaria eradication and elimination: views on how to translate a vision into reality. BMC Med..

[5] Alonso PL, Brown G, Arevalo-Herrera M, Binka F, Chitnis C, Collins F (2011). A research agenda to underpin malaria eradication. PLoS Med..

[6] Tatem AJ, Smith DL, Gething PW, Kabaria CW, Snow RW, Hay SI (2010). Ranking of elimination feasibility between malaria-endemic countries. Lancet.

[7] Feachem RGA, Phillips AA, Hwang J, Cotter C, Wielgosz B, Greenwood BM (2010). Shrinking the malaria map: progress and prospects. Lancet.

[8] Gething PW, Elyazar IRF, Moyes CL, Smith DL, Battle KE, Guerra CA (2012). A long neglected world malaria map: *Plasmodium vivax* endemicity in 2010. PLoS Negl Trop Dis..

[9] Beeson JG, Chu CS, Richards JS, Nosten F, Fowkes FJI (2015). *Plasmodium vivax* malaria: challenges in diagnosis, treatment and elimination. Pediatr Infect Dis J..

[10] Guerra CA, Howes RE, Patil AP, Gething PW, Van Boeckel TP, Temperley WH (2010). The international limits and population at risk of *Plasmodium vivax* transmission in 2009. PLoS Negl Trop Dis..

[11] Battle KE, Gething PW, Elyazar IRF, Moyes CL, Sinka ME, Howes RE (2012). The global public health significance of *Plasmodium vivax*. Adv Parasitol.

[12] Baird JK (2013). Malaria caused by *Plasmodium vivax*: recurrent, difficult to treat, disabling, and threatening to life—averting the infectious bite preempts these hazards. Pathog Glob Health..

[13] Mueller I, Galinski MR, Baird JK, Carlton JM, Kochar DK, Alonso PL (2009). Key gaps in the knowledge of *Plasmodium viva*x, a neglected human malaria parasite. Lancet Infect Dis..

[14] Howes RE, Battle KE, Mendis KN, Smith DL, Cibulskis RE, Baird JK (2016). Global epidemiology of *Plasmodium vivax*. Am J Trop Med Hyg.

[15] Genton B, D’Acremont V, Rare L, Baea K, Reeder JC, Alpers MP (2008). *Plasmodium vivax* and mixed infections are associated with severe malaria in children: a prospective cohort study from Papua New Guinea. PLoS Med..

[16] White NJ, Pukrittayakamee S, Hien TT, Faiz MA, Mokuolu OA, Dondorp AM (2014). Malaria. Lancet.

[17] Mekonnen SK, Aseffa A, Medhin G, Berhe N, Velavan TP (2014). Re-evaluation of microscopy confirmed *Plasmodium falciparum* and *Plasmodium vivax* malaria by nested PCR detection in southern Ethiopia. Malar J..

[18] Ashton RA, Kefyalew T, Rand A, Sime H, Assefa A, Mekasha A (2015). Geostatistical modeling of malaria endemicity using serological indicators of exposure collected through school surveys. Am J Trop Med Hyg.

[19] Stevenson JC, Stresman GH, Baidjoe A, Okoth A, Oriango R, Owaga C (2015). Use of different transmission metrics to describe malaria epidemiology in the highlands of western Kenya. Malar J..

[20] Alemu A, Muluye D, Mihret M, Adugna M, Gebeyaw M (2012). Ten year trend analysis of malaria prevalence in Kola Diba, North Gondar. Northwest Ethiopia. Parasit Vectors..

[21] Ayalew S, Mamo H, Animut A, Erko B (2016). Assessment of current malaria status in light of the ongoing control interventions, socio-demographic and environmental variables in Jiga Area, Northwest Ethiopia. PLoS ONE..

[22] Woyessa A, Deressa W, Ali A, Lindtjørn B (2012). Prevalence of malaria infection in Butajira area, south-central Ethiopia. Malar J..

[23] Alemu A, Abebe G, Tsegaye W, Golassa L (2011). Climatic variables and malaria transmission dynamics in Jimma town, South West Ethiopia. Parasit Vectors..

[24] Getachew S, To S, Trimarsanto H, Thriemer K, Clark TG, Petros B (2015). Variation in complexity of infection and transmission stability between neighbouring populations of *Plasmodium vivax* in Southern Ethiopia. PLoS ONE.

[25] Ashton RA, Kefyalew T, Tesfaye G, Pullan RL, Yadeta D, Reithinger R (2011). School-based surveys of malaria in Oromia Regional State, Ethiopia: a rapid survey method for malaria in low transmission settings. Malar J..

[26] Jima D, Getachew A, Bilak H, Steketee RW, Emerson PM, Graves PM (2010). Malaria indicator survey 2007, Ethiopia: coverage and use of major malaria prevention and control interventions. Malar J..

[27] Abeku TA, Helinski MEH, Kirby MJ, Kefyalew T, Awano T, Batisso E (2015). Monitoring changes in malaria epidemiology and effectiveness of interventions in Ethiopia and Uganda: beyond Garki Project baseline survey. Malar J..

[28] Khaireh BA, Briolant S, Pascual A, Mokrane M, Machault V, Travaillé C (2012). *Plasmodium vivax* and *Plasmodium falciparum* infections in the Republic of Djibouti: evaluation of their prevalence and potential determinants. Malar J..

[29] Molla E, Ayele B (2015). Prevalence of malaria and associated factors in Dilla town and the surrounding rural areas, Gedeo Zone, Southern Ethiopia. J Bacteriol Parasitol..

[30] Alemu A, Tsegaye W, Golassa L, Abebe G (2011). Urban malaria and associated risk factors in Jimma town, south-west Ethiopia. Malar J..

[31] Graves PM, Richards FO, Ngondi J, Emerson PM, Shargie EB, Endeshaw T (2009). Individual, household and environmental risk factors for malaria infection in Amhara, Oromia and SNNP regions of Ethiopia. Trans R Soc Trop Med Hyg.

[32] Haji Y, Fogarty AW, Deressa W (2016). Prevalence and associated factors of malaria among febrile children in Ethiopia: a cross-sectional health facility-based study. Acta Trop.

[33] Ghebreyesus TA, Haile M, Witten KH, Getachew A, Yohannes AM, Yohannes M (1999). Incidence of malaria among children living near dams in northern Ethiopia: community based incidence survey. BMJ.

[34] Peterson I, Borrell LN, El-Sadr W, Teklehaimanot A (2009). A temporal-spatial analysis of malaria transmission in Adama, Ethiopia. Am J Trop Med Hyg..

[35] Lautze J, McCartney M, Kirshen P, Olana D, Jayasinghe G, Spielman A (2007). Effect of a large dam on malaria risk: the Koka reservoir in Ethiopia. Trop Med Int Health..

[36] Loha E, Lunde TM, Lindtjørn B (2012). Effect of bednets and indoor residual spraying on spatio-temporal clustering of malaria in a village in south Ethiopia: a longitudinal study. PLoS ONE.

[37] Ayele DG, Zewotir TT, Mwambi HG (2012). Prevalence and risk factors of malaria in Ethiopia. Malar J..

[38] Peterson I, Borrell LN, El-Sadr W, Teklehaimanot A (2009). Individual and household level factors associated with malaria incidence in a highland region of Ethiopia: a multilevel analysis. Am J Trop Med Hyg.

[39] Woyessa A, Deressa W, Ali A, Lindtjørn B (2013). Malaria risk factors in Butajira area, south-central Ethiopia: a multilevel analysis. Malar J..

[40] Sena L, Deressa W, Ali A (2014). Dynamics of *Plasmodium falciparum* and *Plasmodium vivax* in a micro-ecological setting, Southwest Ethiopia: effects of altitude and proximity to a dam. BMC Infect Dis.

[41] Siri JG (2014). Independent associations of maternal education and household wealth with malaria risk in children. Ecol Soc..

[42] Loha E, Lindtjørn B (2012). Predictors of *Plasmodium falciparum* malaria incidence in Chano Mille, South Ethiopia: a longitudinal study. Am J Trop Med Hyg.

[43] Loha E, Tefera K, Lindtjørn B (2013). Freely distributed bed-net use among Chano Mille residents, south Ethiopia: a longitudinal study. Malar J..

[44] WHO (1991). Basic Malaria Microscopy.

[45] Douglas NM, Nosten F, Ashley EA, Phaiphun L, van Vugt M, Singhasivanon P (2011). *Plasmodium vivax* recurrence following falciparum and mixed species malaria: risk factors and effect of antimalarial kinetics. Clin Infect Dis.

[46] Looareesuwan S, White N, Bunnag D, Chittamas S, Harinasuta T (1987). High rate of *Plasmodium vivax* relapse following treatment of falciparum malaria in Thailand. Lancet.

[47] White NJ (2011). Determinants of relapse periodicity in *Plasmodium vivax* malaria. Malar J..

[48] White NJ, Imwong M (2011). Relapse. Adv Parasitol.

[49] Mueller I, Galinski MR, Tsuboi T, Arevalo-Herrera M, Collins WE, King CL (2013). Natural acquisition of immunity to *Plasmodium vivax*: epidemiological observations and potential targets. Adv Parasitol.

